# Epithelial–mesenchymal transition and its transcription factors

**DOI:** 10.1042/BSR20211754

**Published:** 2021-12-23

**Authors:** Pallabi Debnath, Rohit Singh Huirem, Paloma Dutta, Santanu Palchaudhuri

**Affiliations:** Amity Institute of Biotechnology, Amity University Kolkata, West Bengal, India

**Keywords:** Epithelial mesenchymal transition, gene expression regulation, Transcription factor, transcriptional regulatory network

## Abstract

Epithelial–mesenchymal transition or EMT is an extremely dynamic process involved in conversion of epithelial cells into mesenchymal cells, stimulated by an ensemble of signaling pathways, leading to change in cellular morphology, suppression of epithelial characters and acquisition of properties such as enhanced cell motility and invasiveness, reduced cell death by apoptosis, resistance to chemotherapeutic drugs etc. Significantly, EMT has been found to play a crucial role during embryonic development, tissue fibrosis and would healing, as well as during cancer metastasis. Over the years, work from various laboratories have identified a rather large number of transcription factors (TFs) including the master regulators of EMT, with the ability to regulate the EMT process directly. In this review, we put together these EMT TFs and discussed their role in the process. We have also tried to focus on their mechanism of action, their interdependency, and the large regulatory network they form. Subsequently, it has become clear that the composition and structure of the transcriptional regulatory network behind EMT probably varies based upon various physiological and pathological contexts, or even in a cell/tissue type-dependent manner.

## Introduction

**Figure 1:**
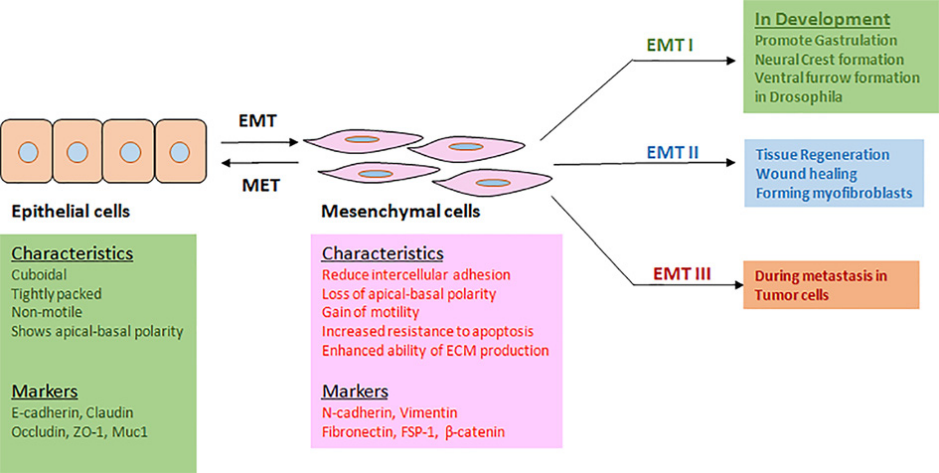
EMT: characteristics, markers, and contexts EMT is activated in different physiological and pathological contexts, thereby facilitating cellular movement. It is associated with both morphological and characteristics’ changes.

Epithelial–mesenchymal transition or EMT is a biological process by which cuboidal, tightly packed, and non-motile epithelial cells adopt a loosely organized mesenchymal or fibroblast-like phenotype with properties such as reduced intercellular adhesion, loss of apical–basal polarity, gain of motility and invasive ability, increased resistance to apoptosis, and enhanced ability of ECM production (Figure 1). EMT (type I) was originally identified as a critical program during early embryonic morphogenesis and was found to be involved in various developmental stages such as gastrulation, neural crest formation, and heart morphogenesis. EMT (type II) was found to be induced in response to inflammation, for example during wound healing, tissue regeneration, and fibrosis. EMT (type III) program was shown to be activated during metastasis, which is the primary cause of mortality in cancer patients [1–5]. Significantly, EMT has been found to induce other properties such as, acquisition of stem cell-like phenotype, resistance to chemotherapy, immune-evasion etc in cancer cells, thus making these cells difficult to eradicate. Complexity of the EMT mechanism is also exemplified by the fact that cells undergoing EMT have sometimes been observed to attain a hybrid epithelial–mesenchymal phenotype, expressing both epithelial and mesenchymal markers.

### EMT in development

The EMT (type I) was originally identified as a critical program during early embryonic morphogenesis and was found to be involved at various early developmental stages [4,6,7]. Indeed, multiple rounds of EMT and its reverse process Mesenchymal–Epithelial Transition or MET, has been shown to be essential for the development of the complex three-dimensional structure of the internal organs. Accordingly, these EMTs are referred to as primary, secondary, and tertiary EMT. The primary EMTs include those involved during mammalian implantation, metazoan gastrulation, and neural crest formation in vertebrates. Gastrulation is a process through which the cells in the bilaminar embryonic disc of blastula/blastocyst move and rearrange to form the gastrula with three germ layers namely endoderm, mesoderm, and ectoderm. EMT is one of the mechanisms activated during gastrulation through which cells get separated from the epiblast layer and migrate into a specific region within embryo such as the primitive streak in amniotes, the vegetal pole in sea urchin or the ventral furrow in *Drosophila*, and thereby form the three germ layers at a defined location within the embryo.

Following gastrulation, the cuboidal epithelial cells in the ectoderm above the mesoderm change into columnar epithelial cells which form the neural plate and become distinguishable from the pre-epidermal cells surrounding them. The changes in cell shape and adhesion properties lead to bending of the neural plate leading to the formation of neural tube, which further develops into the central nervous system. During this process, neural crest is formed between epidermal layer and the neural tube. The neural crest cells then undergo EMT within the dorsal neural epithelium and migrate to their target sites, where they differentiate into different derivatives such as most components of the peripheral nervous system including neurons and glial cells, melanocytes, endocrine cells, and craniofacial structures [8–11].

Notably, these primary EMT events are followed by a set of differentiation events, that generate different types of cells, which undergo MET and acquire transient epithelial structures such as notochord, somites, precursors of the urogenital system, and the somatopleure and splanchopleure. Except for notochord, all these secondary epithelia undergo a secondary EMT in presence of external signals from their microenvironment and generate mesenchymal cells with more restricted differentiation potential. Tertiary EMT can be observed during cushion mesenchyme formation from the atrioventricular canal or outflow tract in the heart. Together, all these sequential EMT and MET events lead to the development of a fully functional embryo.

### EMT in pathogenesis

Interestingly, EMT (type II) was also found to be induced in response to inflammation, for example during wound healing, tissue regeneration, and fibrosis [1,4,6,12]. EMT-like event has been found to occur during wound healing, where keratinocytes at the border of the wound undergo partial EMT and acquire a metastable state. This allows the keratinocytes to move while maintaining loose contact with the surroundings. In case of tissue fibrosis, the myofibroblasts accumulate and secrete a large amount of collagen which is deposited as fibers. This compromises the organ function and ultimately leads to its failure. It was found that a significant portion of the myofibroblasts is generated through the conversion of epithelial cells by the EMT process. Infact, the lens epithelium, endothelium, hepatocytes, as well as cardiomyocytes were all shown to undergo EMT and facilitate the tissue fibrosis process.

EMT (type III) program has further been found to be activated during metastasis, which is the primary cause of mortality in cancer patients [2,6,7]. A small population of cancer cells in the primary tumor activates the EMT program to gain motility and invasiveness by which they disseminate from their site of origin, released into the circulation, and move to a distant site. Small aggregates of tumor cells extending or detaching from the bulk tumor and entering the adjacent stroma have been detected at the invasive fronts of human tumors such as colon carcinoma, breast carcinoma, papillary thyroid carcinoma, cervical carcinoma etc. This was found to be concomitant with reduced expression of E-cadherin, selective loss of basement membrane and/or increased expression of Vimentin etc. Interestingly enough, it was found that often cancer cells undergoing EMT do not show a complete conversion, rather they pass through EMT at different extents. Consequently, some of these cells may express both epithelial and mesenchymal markers, thereby exhibiting a hybrid epithelial–mesenchymal state. Nonetheless, some of these cells might also show complete conversion exhibiting only mesenchymal phenotype and respective markers. One reason for this apparent incomplete conversion could be the difficulty to distinguish fully converted mesenchymal cells originated from epithelial cancer cells via EMT from stromal cells or tumor-associated fibroblasts. Significantly, EMT, especially partial EMT has been found to induce stem cell-like properties such as self-renewal ability and enhanced differentiation potential in cancer cells. This provides the cancer cells a tremendous advantage to survive and sustain inside the host.

## Master regulators of EMT

**Figure 2:**
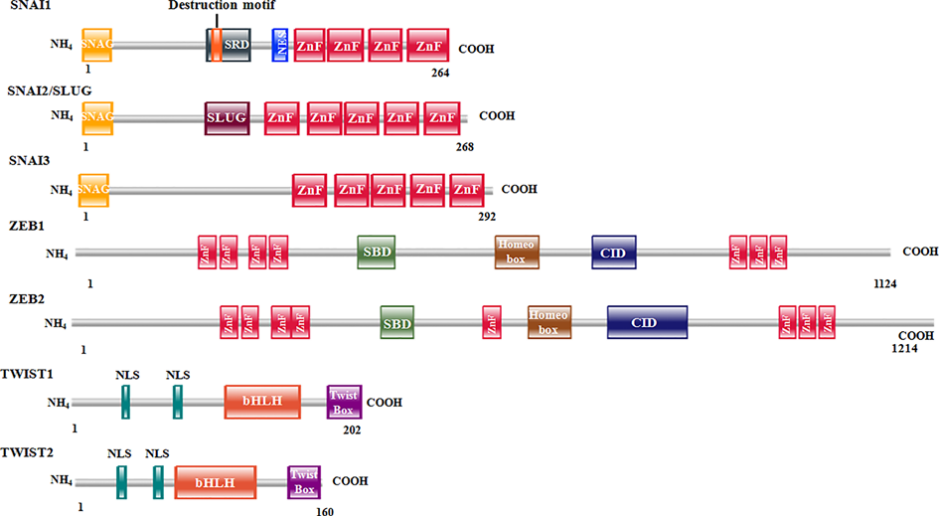
Structure of master regulators of EMT Schematic depiction of EMT master regulators and their respective domains with their comparative size; ZEB2 being the largest and TWIST2 being smallest. SNAI1 and SNAI2 have zinc fingers in their C-terminus, whereas ZEB1 and ZEB2 have zinc fingers on both sides.

**Figure 3:**
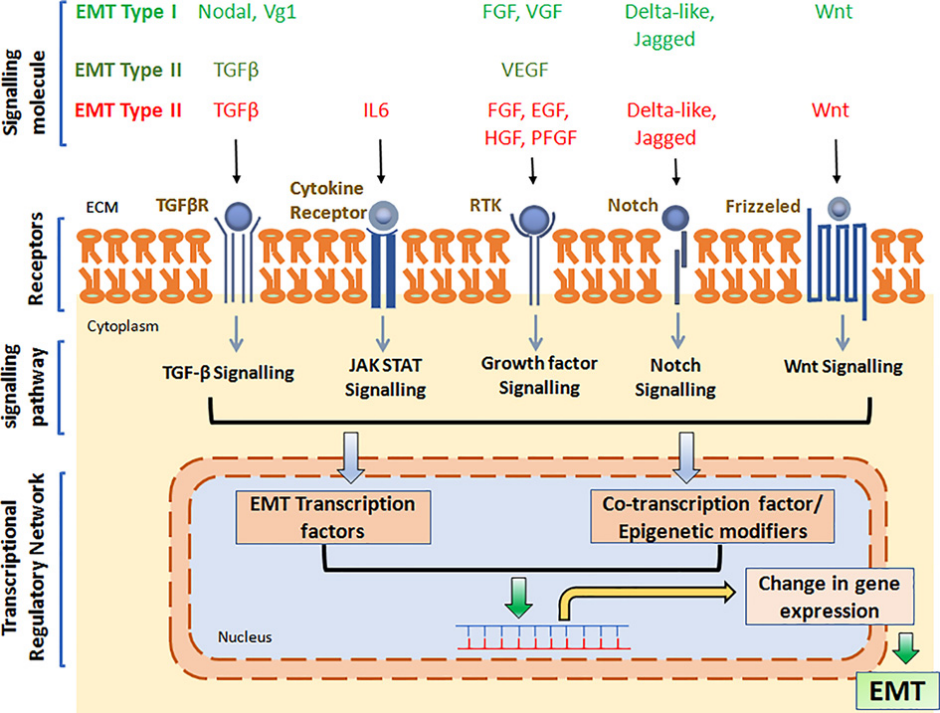
EMT overview EMT is induced by a variety of signaling molecules, which stimulate cognate receptors on the cell surface and thereby activate downstream signaling cascade, leading to activation of EMT TFs and associated co-regulators and epigenetic regulators. This subsequently turns ‘on’ or ‘off’ specific genes. The altered transcriptome and proteome further supports the transition.

The EMT process under different contexts were found to be activated by several signaling molecules. For example, EMT associated with gastrulation is activated by the canonical Wnt signaling pathway, the TGFβ superfamily proteins Nodal and Vg1 and growth factors such as FGF, EGF etc. During neural crest formation, EMT is induced by signaling molecules such as Wnt, BMP, FGF, Notch etc. Type II EMT is induced by factors such as VEGF and TGFβ, whereas type III EMT, or EMT associated with metastasis is induced by a large set of signaling molecules such as Wnt, TGFβ, BMP, FGF, EGF, HGF, PDGF, VEGF, Estrogen, SCF etc. Collectively, these molecules stimulate various signaling pathways, thereby activating a small set of transcription factors (TFs) or master regulators of EMT. These include Snail Family proteins Snail1 (Snail), Snail2 (Slug), Zinc finger E-box binding (Zeb) homeobox family proteins Zeb1 and Zeb2, and TWIST family proteins Twist1 and Twist2 (Figure 2). Together, these TFs act to suppress expression of epithelial markers such as E-cadherin, Claudin, Occludin, Mucin-1, PTEN, RKIP etc. as well as activate mesenchymal markers such as N-cadherin, Vimentin, Vitronectin, Matrix Metalloproteases etc. (Figure 3 and Table 1). E-cadherin (CDH1), a calcium-dependent cell adhesion protein and part of the cell–cell adheren junction, is one of the most studied genes regulated by several EMT TFs. Down-regulation of CDH1 by the EMT TFs has been shown to increase cancer cell proliferation, invasiveness, and/or metastasis. Expression of various components of tight junction such as Claudins and Occludins, as well as gap junction components are also regulated by EMT TFs. Decreased expression of all these junctional proteins by EMT TFs lead to reduced cell–cell attachment and facilitate the transition. EMT TFs have also been shown to up-regulate expression of various mesenchymal markers such as N-cadherin (CDH2), Vimentin, Fibronectin, and matrix metalloproteinases, which together facilitate the mesenchymal cell adhesion, migration, and invasion. In the following section as well as in Table 1, we have discussed the master TFs of EMT, their function as well as the target genes (direct) they regulate.

**Table 1:** Master regulators of EMT

EMT TFs	Effect on EMT (cell/tissue types tested)			Direct target genes (cell/tissue types tested)
	EMT I	EMT II	EMT III	
SNAI1	Inducer (gastrulation, neural crest development etc.)	Inducer (canine kidney cells, adult kidney fibrosis, human corneal endothelium)	Inducer (colon carcinoma cells, breast cancer, non-small cell lung cancer, oral squamous cell carcinoma, head and neck cancer, hypopharyngeal carcinoma)	CDH1 (kidney cells, breast epithelial cells), SNAI1 (colon cancer cells), Claudin1 (kidney cells), Claudin 7 and Occludin (*in vitro* and kidney cells), ZEB1 and MMP9 (hepatoma cells), Vimentin (breast epithelial cells), Fibronectin (colon cancer cells, breast epithelial cells), Twist1 (breast epithelial cells), SNAI2 (ovarian cancer cells)
SNAI2/SLUG	Inducer (gastrulation, neural crest development etc.)	Inducer (canine kidney cells, keratinocytes)	Inducer (melanoma, colon carcinoma cells)	CDH1 (kidney cells, breast epithelial cells), ZEB1 (melanoma), Claudin1 (kidney cells)
ZEB1	Inducer (gastrulation, neural crest development etc.)	Inducer (human alveolar epithelial type II cells, human corneal endothelium, cardiac fibroblasts, lung fibrosis, hepatic stellate cells)	Inducer (melanoma, breast cancer cells, lung cancer cells, pancreatic cancer cells, ameloblastic carcinoma, colorectal cancer cells)	CDH1 (breast cancer cells, pancreatic cancer cells), SETD1B (colon cancer cells), ESRP1 (lung cancer cells), Crumbs3, PATJ, Epcam, Elk3 and Plakophilin 3 (breast cancer cells), OVOL2 (breast epithelial cells)
ZEB2	Inducer (gastrulation, mesoderm development, neural crest formation)	Inducer (cardiac fibrosis)	Inducer (colon cancer cells, breast cancer cells; ovarian, gastric and pancreatic cancer)	CDH1, Plakophilin2, ZO-3, Connexin26 (colon cancer cells), Rab25 (breast cancer cells)
TWIST1	Inducer (gastrulation, mesoderm development, neural crest development)	Inducer (kidney, lung and skin fibrosis)	Inducer (breast cancer, colon cancer, prostate cancer)	SNAI1 (palatal shelves), SLUG (breast epithelial cells), ZEB1 (colon cancer cells), CDH1 (breast cancer cells), CDH2 (breast and prostate cancer cells)

### Snail family TFs

Snail was first identified in *Drosophila melanogaster* [13], and was found to be essential for mesoderm formation during gastrulation [14]. Subsequently, presence of two additional Snail family proteins, namely escargot and worniu, were established in the fruit fly. In invertebrates, a single Snail family protein was found, whereas, in vertebrates, three members of the Snail family proteins have been reported, namely, Snail1 (Snail), Snail2 (Slug), and Snail3 (Smuc). In invertebrates such as sea urchin, Snail has been found to suppress expression of E-cadherin (CDH1) and induce delamination of primary mesenchyme cells via EMT. Snail was also found to be critical for fly gastrulation. In vertebrates, during gastrulation, Snail genes are induced by TGFβ family of proteins, while their expression is maintained by FGF. Indeed, mouse embryos deficient in Snail genes (−/−) fail to gastrulate and exhibit defective mesoderm germ layer formation [15]. Notably, E-cadherin expression is retained in the mesoderm of these embryos, suggesting incomplete EMT. In contrast, Snail2 null (−/−) mice exhibited no EMT failure [16]. In chicken, Snail2 is expressed in the primitive streak and its perturbation does lead to a gastrulation phenotype [17].

Activation of Snail1 alone was found to disrupt tissue homeostasis and stimulate adult kidney fibrosis [18]. Snail was shown to trigger the EMT process leading to repression of E-cadherin, Renin, HNF-1β and increased expression of Snail2, Vimentin, SMA, Collagen-I etc., thereby converting epithelial cells into myofibroblasts. Notably, high level of Snail1 was found in patients’ fibrotic kidney. Snail2 was found to be expressed in keratinocytes at the migratory front of wound and perturbation of its expression was found to affect the wound healing process [19].

Significantly, expression of Snail family TFs such as Snail and Slug, have been found to correlate positively with reduced E-cadherin expression, increased invasiveness, dedifferentiation status, and aggressiveness in tumor specimens obtained from patients with breast, gastric, colon and hepatocellular carcinoma (HCC), and synovial sarcoma [20–27]. Snail1 was found to be critical for the tumorigenesis and lymph node metastasis of human breast cancer cell line MDA-MB231 as well [28]. Furthermore, Snail1 suppression was shown to inhibit lung cancer cell migration, tumor growth, and metastasis both *in vitro* and *in vivo* [29]. Snail knockdown was also reported to reduce cell motility and stemness of ovarian cancer cells, which in turn reduced the tumor burden in orthotopic xenograft mouse model [30]. Not surprisingly therefore, Snail was shown to activate EMT in a wide variety of cell lines including cancer cells [29,31–39]. Snail1 was further reported to directly regulate expression of many EMT-associated genes such as *CDH1* [31,34,40–42], *SNAI1* [43], *Claudin1* [42,44], *Claudin 7* and *Occludin* [45], *ZEB1* and *MMP9* [46], *Vimentin* [34], *Fibronectin* [47], *Twist1* [48,49], and *SNAI2* [50]. SNAI2/Slug was also shown to induce EMT in various cells lines and directly regulate EMT-associated gene expression [42,44,51–53].

It should be noted that all the Snail family members were found to carry a highly conserved C-terminal domain consisting of four to six C2H2-type zinc fingers through which they bind to the E-Box motif, 5′-CANNTG-3′, present in the target gene promoters. Furthermore, the N-terminal domain of vertebrate Snail family members carry a conserved SNAG domain through which they interact with and therefore recruit the Polycomb Repressive Complex (PRC) 2 (PRC2) containing a wide variety of transcriptional co-repressor complexes to the target gene promoters [54,55]. The repressor complex in turn induces H3/H4 deacetylation, H3K4 demethylation and H3K9 and H3K27 hypermethylation, followed by increased DNA methylation through recruitment of DNMTs at the target gene promoter regions. Collectively, all these epigenetic modifications create a state of closed chromatin structure at the target gene promoters, thereby causing silencing of Snail target genes. Interestingly, the Snail family members were also reported to activate target gene transcription by directly binding to the promoter and/or enhancer elements of the target genes in cooperation with different transcriptional activators. It has been shown that the CREB-binding protein, CBP, interacted with Snail and acetylated Lys^146^ and Lys^187^ residues, thereby inhibiting the formation of the repressor complex [56]. Snail was also found to collaborate with EGR-1 and SP1 to directly bind the MMP9 and Zeb1 promoter and activate their transcription [46].

### Zeb family TFs

The Zeb family of transcriptional regulators consists of two members, Zeb1 (also known as TCF-8 or δEF1) and Zeb2 (also named SIP1). Zeb1 expression during development was found to be inversely proportional to E-cadherin expression in cells of mesoderm origin such as notochord, somites etc. as well as in neural crest derivatives [57]. Zeb1 knockout (−/−) mice die shortly after birth and show severe T-cell deficiency of the thymus as well as skeletal defects of various lineages indicating critical role of the protein during embryogenesis [58]. Zeb2 was also found to play essential roles during embryogenesis, such as during gastrulation in chicken [59], mesodermal development [60], and neural crest development in mice [61]. Zeb2 knockout mice was found to be embryonically lethal with signs of abnormal development of the nervous system [61,62]. It was reported that overexpression of Zeb1 was sufficient to reduce expression of both E-cadherin and p63 and simultaneously enhance vimentin expression in MCF-10A cells. Significantly, increased expression of either of Zeb1 and/or Zeb2 has been found to be associated with poor clinical outcome in various cancer types including breast, colorectum, pancreas, ovarian etc [63–67]. Subsequently, the Zeb family TFs were reported to induce EMT in various cell lines including cancer cells and directly regulate expression of several EMT-associated genes [68–78].

Zeb1 has also been shown to play critical role in promoting fibrosis [79] in human corneal endothelial cells, along with Snail1 [80] as well as in cardiac fibroblasts [81]. Zeb1-mediated paracrine signaling was further shown to induce intestinal lung fibrosis by facilitating the development of profibrogenic microenvironment [82]. Moreover, Zeb1 has been shown to contribute to the profibrotic process by activating the hepatic stellate cells [83]. The other member of the Zeb family, Zeb2, has also been shown to be involved in cardiac fibrosis by facilitating fibroblast to myofibroblast conversion [84].

Both Zeb1 and Zeb2 belong to C2H2 type zinc finger family proteins with a centrally located homeodomain as well as four N-terminal zinc fingers and three C-terminal zinc fingers [85]. Both Zeb1 and Zeb2 were found to bind to the E-box consensus sequence 5′-CANNTG-3′ on the CDH1 promoter and suppress its expression through recruitment of repressor protein complex involving C-terminal-binding protein (CtBP), Polycomb proteins and CoREST, as well as through recruitment of SWI/SNF chromatin remodeling complex BRG1 [68,69,73,86]. Moreover, Zeb1 was also found to form a repressor complex with Sirt-1 (a class-III histone deacetylase (HDAC)), which binds the promoter region of CDH1 and suppresses its expression in prostate and pancreatic cancer cells undergoing EMT [87,88]. Significantly, apart from its role as transcriptional repressor, Zeb1 was also found to act as a transcriptional activator, thus inducing the expression of mesenchymal cell-specific genes such as collagen, smooth muscle actin, genes in the vitamin D signaling pathway etc [89–95]. Zeb1 was shown to bind activated Smads as well as the Histone Acetyltransferase p300. These binding abilities of Zeb1 facilitate Smad–p300 complex formation as well as dissociation of Zeb1 from its co-repressor CtBP, thus converting Zeb1 from a transcriptional repressor into a transcriptional activator [92–94]. Recently, ZEB1 was shown to interact with AP-1 factors FOSL1 and Jun as well as the Hippo pathway effector YAP to form a multimeric transactivation complex, which in turn activated tumor-promoting genes in breast cancer cells [96]. Zeb1 was also shown to promote EMT by directly regulating expression of SETD1B (Lysine Methyltransferase induced active epigenetic marks), ESRP1 (epithelial cell-specific splicing regulatory protein), Crumbs3 (involved in epithelial cell polarity as well as tight junction morphogenesis), PATJ (forms part of tight junction as well as epithelial cell polarity), Epcam (epithelial cell adhesion molecule), Plakophilin3 (involved in desmosome-dependent cell adhesion and signaling) etc. Zeb2 was further shown to directly regulate various epithelial-specific junctional proteins such as CDH1, Plakophilin2 (desmosome), ZO3 (tight junction), and Connexin26 (gap junction) (Table 1).

### bHLH family TFs: twist family proteins

The Twist family transcriptional regulators Twist1 and Twist2 belongs to the basic–helix–loop–helix (bHLH) family of proteins. Twist1 was first identified in drosophila, where it was shown to be critical for the embryogenesis process. Twist1 null embryo showed abnormal gastrulation with no mesoderm and failed to survive with a ‘twisted’ appearance [97,98]. The other member of the Twist family, Twsit2 has also been found to be important for embryogenesis. Significantly, Twist was found to regulate the transcriptional switching of E- to N-cadherin [99]. Twist was also found to be critical for EMT during embryogenesis in sea urchin embryos as well as in mice. Twist mutation in mice causes failure in cranial neural tube closure, indicating its role in proper migration and differentiation of neural crest and head mesenchymal cells [100,101]. In fact, Twist1-deficient mouse embryos die at approximately E11.5. Twist was also found to promote tumor cell invasion and subsequent metastasis via stimulation of the EMT process. Additionally, Twist1 was shown to play significant role during tissue fibrosis [102,103].

Interestingly, hypoxia or overexpression of HIF-1α was reported to induce EMT via activation of Twist. Subsequently, it was shown that HIF-1α binds directly to the hypoxia-response element at the proximal promoter region of Twist and regulate its expression [104]. Twist1 by itself was able to induce EMT when overexpressed in breast and kidney epithelial cells [105]. Notably, Twist1 was demonstrated to induce N-cadherin transcription by binding to the E-box *cis*-element located within the first intron of the N-cadherin gene in prostate cancer cells [106]. Moreover, it could also bind the E-cadherin promoter directly and repress E-cadherin gene expression [107]. Further study revealed that Twist1 interacted with several components of the Mi2/nucleosome remodeling and deacetylase (Mi2/NuRD) complex such as MTA2, RbAp46, Mi2 and HDAC2, and recruited them to the proximal regions of the E-cadherin promoter for transcriptional repression of the gene [108]. Additionally, methylation of Twist1 by PRMT1 at residue R34 was shown to affect the E-cadherin transcriptional repression [109].

## Other EMT TFs

Significantly, findings from multiple labs over the years have also identified many additional TFs besides the master regulators of EMT, that are found to play significant role during EMT under various contexts. This suggests that EMT is probably regulated by a much larger group of TFs than previously thought. In this section, we shall focus on these ‘other’ DNA-binding EMT TFs and discuss their involvement in EMT including their known direct targets (Table 2).

**Table 2:** Other EMT TFs

EMT TFs	Effect on EMT (cell/tissue types tested)			Direct target genes (cell/tissue types tested)
	EMT I	EMT II	EMT III	
E12/E47	-	Inducer (renal proximal tubular epithelial cells, MDCK cells)	Inducer (colon cancer metastasis)	CDH1 (MDCK cells)
KLF4	-	-	Suppressor (lung epithelial cells, nasopharyngeal carcinoma cells, hepatocellular carcinoma cells, lung cancer cells, human endometrial carcinoma cells, pancreatic cancer cells, colorectal cancer cells)	CDH1 (nasopharyngeal carcinoma cells)
			Reversal of EMT (gastric cancer cells)	Serine/threonine kinase 33 (gastric cancer cells)
KLF8	-	-	Inducer (MDCK, MCF-10A, Panc-1, gastric cancer cell line SGC7901, breast cancer cells)	CDH1 (breast cancer cells)
KLF10	-	-	Suppressor (A549, Panc-1)	SLUG (A549, Panc-1)
FOXC1	-	-	Inducer (esophageal cancer, nasopharyngeal cancer, basal like breast cancer, glioma, cervical cancer, and hepatocellular carcinoma)	FGFR1 (NMuMG cells), ZEB2 (esophageal cancer cells)
FOXC2	-	-	Inducer (mouse mammary carcinoma cell, mammary epithelial cells, basal-type human breast cancer cells, ovarian cancer cells)	ZEB1 (breast cancer cells)
FOXQ1	-	-	Inducer (basal-like breast cancer, Mammary, bladder and colon epithelial cells, gastric cancer cells)	CDH1 (breast cancer cells), CDH2 (breast cancer cells)
FOXK1	-	-	Inducer (colon cancer cells)	ND
FOXG1	-	-	Inducer (human hepatocellular carcinoma cells)	ND
FOXM1	-	-	Inducer (non-small cell lung cancer, kidney cells)	ND
FOXF2	-	-	Suppressor (basal-like breast cancer cells, triple-negative breast cancer cells)	TWIST1, FOXC2, FOXQ1 (basal-like breast cancer cells)
FOXN2	-	-	Suppressor (breast cancer cells)	SLUG (breast cancer cells)
FOXO3a	-	-	Suppressor (prostate cancer cells)	ND
SOX4	-	-	Inducer (mammary epithelial cells, breast cancer cells, triple-negative breast cancer cells, lung carcinoma cells, gastric cancer cells, prostate cancer calls, renal cancer cells)	EZH2 (NMuMG cells), ADAM28 (human breast and lung carcinoma cells), CDH2 (triple-negative breast cancer cells)
SOX9	Inducer (neural crest development)	Inducer (liver fibrosis)	Inducer (thyroid cancer cells, prostate cancer cells, non-small cell lung cancer cells, gastric cancer cells, human oral squamous carcinoma cells, gastric carcinoma cells)	ND
SOX11	-	-	Inducer (breast cancer cells)	SLUG (breast cancer cells)
			Promoted epithelial–mesenchymal hybrid characteristics (ER-negative DCIS.com breast cancer cell)	ND
RUNX1	-	Inducer (renal fibrosis)	Inducer (colorectal cancer cells, kidney epithelial cells)	p110δ (renal tubular epithelial cells)
RUNX2	Inducer (chicken atrioventricular canal)	Inducer (lung fibrosis)	Inducer (thyroid cancer cells, hepatocellular cancer cells, renal cell carcinoma cells, non-small cell lung cancer cells)	ND
GATA4	Inducer of cell migration (gastrulation)	-	Inducer (nasopharyngeal carcinoma cell) Induce moderate MET (hepatocellular carcinoma cells)	SLUG (nasopharyngeal carcinoma cell)
GATA6	Serpent, ortholog of human GATA6 acts as inducer of EMT in *Drosophila* endoderm. Inducer of cell migration (gastrulation)	Inducer (canine kidney cells)	Inducer (cholangiocarcinoma cells, breast cancer cells) Suppressor (pancreatic cancer cells)	MUC1 (cholangiocarcinoma cells), SNAI2 (beast cancer cells), crumbs (*Drosophila* endoderm, canine kidney cells) CDH1 and VIM (pancreatic cancer cells)
WT1	Inducer of MET in early kidney development; Inducer (epicardial cells); suppressor (human adult epicardial cells)	Inducer (lung fibrosis)	Inducer (ovarian cancer cells) Promoted epithelial–mesenchymal hybrid state (clear cell renal cell carcinoma)	SNAI1 (epicardial cells), CDH1 (epicardial cells, ovarian cancer cells), SLUG (epicardial cells)
Goosecoid	Inducer of cell migration (gastrulation)	-	Inducer (breast cancer cells, hepatocellular carcinoma cells)	ND
Six1	-	Inducer (lung epithelial cell fibrosis)	Inducer (mammary carcinoma cells, colorectal cancer cells, cervical cancer cells, lung epithelial cells, immortalized human keratinocytes)	ND
Prrx1	Inducer (chicken embryo)	Inducer (hepatic fibrosis, canine kidney cells)	Inducer (gastric cancer cells, non-small cell lung cancer cells, salivary adenoid cystic carcinoma cells)	ND
			Suppressor (lung cancer cells)	ND
Elk3	-	Inducer (liver fibrosis)	Inducer (breast cancer cells)	ND
Brachyury	Required for mesoderm formation as well as cell movement during gastrulation	Inducer (kidney fibrosis)	Inducer (pancreatic cancer cell line, lung carcinoma cells, oral squamous carcinoma cells, kidney cells)	ND
FOSL1	-	-	Inducer (prostate cancer cells, non-small cell lung cancer cells, mammary epithelial cells)	TGFB1, ZEB1, ZEB2 (mammary epithelial cells)
FOSL2	-	-	Inducer (prostate cancer cells, non-small cell lung cancer cells)	ND
JunB	-	Inducer (kidney fibrosis)	Inducer (mammary epithelial cells, uveal melanoma cells)	ND
OVOL1	-	-	Suppressor (prostate cancer cells, triple negative breast cancer cell)	ND
OVOL2	Inducer of MET (fibroblasts)		Suppressor (prostate cancer cells, triple negative breast cancer cell)	ZEB1 (prostate cancer cells)
ALX1	-	-	Inducer (ovarian cancer cells, breast epithelial cells)	ND
ZBTB38	-	-	Inducer (bladder cancer cells)	ND
TFAP2A	-	Positive regulator (human ventricular fibroblasts)	Suppressor (breast epithelial cells)	ZEB2 (breast epithelial cells)
BACH1	-	Inducer (lung fibrosis)	Inducer (esophageal squamous cell carcinoma, pancreatic cancer metastasis)	CDH2, SNAI2, Vimentin, VEGFC (esophageal squamous cell carcinoma)

### E2A proteins: E12/E47 TFs

E12 and E47, members of the class I bHLH TFs, and splice variants of the gene *E2A/TCF3* were shown to induce EMT in human renal proximal tubular cells and in MDCK cells [110–112]. Furthermore, it was demonstrated that E47 can directly bind to E-box element at the E-Cadherin promoter and suppress its expression. Additionally, Zhu et al. showed that the p21-activated kinase 5 (PAK5) phosphorylates E47 at S39 at the cytosol and promotes its entry into the nucleus in an Importin-α-dependent manner. This phosphorylation-induced nuclear entry was found to be critical for E47 to promote EMT and colon cancer metastasis [113].

### Krüppel-like factor family of TFs

Krüppel-like factor 4 (KLF4) has been shown to act as a negative regulator of EMT or even inducer of MET in a wide variety of cell/tissue types by different laboratories [114–123]. In fact, KLF4 expression was found to be down-regulated in cells undergoing EMT in presence of TGFβ. Subsequently, Li et al. have shown that KLF4 can directly bind to CDH1 promoter and activate its transcription [120]. KLF4 mediated direct promoter binding and suppression of Serine/Threonine kinase 33 (STK33) was further shown to cause reversal of EMT [121].

KLF8 has been shown to induce EMT in MDCK, MCF-10A and pancreatic cancer cell line Panc-1 [124,125]. In fact, its expression was found to be enhanced in gastric cancer cell line SGC7901 undergoing EMT in presence of TGFβ [126]. Moreover, silencing of Klf8 was shown to inhibit induction of EMT in this cell line when exposed to TGFβ. Mechanistically, Klf8 was reported to bind directly to the CDH1 promoter at a site distinct from E-boxes and suppress its expression [124].

KLF10, another Krüppel-like factor family protein, has been reported to suppress TGFβ-induced EMT in A549 and Panc-1 cell lines. It was further shown to bind to the SNAI2 (Slug) promoter, recruit HDAC1, and repress its expression [127]. Significantly, *Klf10*-deficient mice showed increased incidence of lung tumor formation as well as increased tumor size compared with wildtype mice, when exposed to 7,12-dimethylbenz(a)anthracene (DMBA).

### Forkhead box family of TFs

The Forkhead box family member FOXC1 expression was elevated in mammary epithelial cells undergoing EMT in presence of TGFβ. FOXC1 was shown to directly bind an upstream regulatory region of the FGFR1 gene, a member of the Fibroblast Growth Factor Receptor family and promote isoform switching. FGFR1 has been shown to induce EMT in urothelial carcinoma cells [128]. Knockdown experiments revealed a regulatory role for FOXC1 in cell migration and invasion. [129]. Furthermore, FOXC1 has been reported to play significant role during EMT in a variety of cancers such as esophageal cancer, nasopharyngeal cancer, basal-like breast cancer, glioma, cervical cancer, and HCC [130–134]. Mechanistically, FOXC1 was shown to stimulate EMT in esophageal cancer cells by binding to the Zeb2 promoter directly in a pre-B-cell leukemia homeobox 1 (PBX1)-dependent manner and thereby induce its expression [131].

Another member of the Forkhead family of TFs, FOXC2 was shown to play a key role during EMT. Elevated level of FOXC2 was found in mouse mammary carcinoma cells undergoing EMT in presence of TGFβ. Ectopic expression of master regulators of EMT such as Snail and Twist, has also been shown to induce FOXC2 expression. Subsequently, FOXC2 was found to be required to induce mesenchymal characteristics as a part of the EMT program [135,136]. Significantly, overexpression and knockdown studies have further showed that FOXC2 is essential for the stem cell characteristics linked with EMT in mammary epithelial cells [136]. All these experimental observations suggest a crucial role for FOXC2 in tumorigenesis and/or metastasis process. Indeed, its expression was found to be significantly correlated with highly aggressive basal type human breast cancer [135]. Additionally, FOXC2 was also shown to be a critical regulator of EMT in ovarian cancer cells as well [137]. Mechanistically, p38-mediated phosphorylation of FOXC2 at S367 residue was found to be essential for the EMT process. The phosphorylated form of FOXC2 was shown to bind to the Zeb1 promoter and regulate its expression, thereby, regulating the EMT, and subsequently, metastasis [138].

Expression of FOXQ1 has been found to be significantly correlated with highly aggressive basal-like breast cancers with poor clinical outcome [139]. Indeed, increased level of FOXQ1 was shown to be important for TGFβ-induced EMT and its associated characteristics in mammary, bladder, and colon epithelial cells [139–141]. Additionally, FOXQ1 was also found to regulate EMT in gastric cancer cells. Not surprisingly therefore, high expression level of FOXQ1 was observed to be associated with poor prognosis in gastric cancer patients [142]. Subsequently, FOXQ1 was shown to bind to the E-cadherin and the N-cadherin promoter directly and regulate their expression [139,140,143].

FOXK1 was shown to be up-regulated in colon cancer cells undergoing EMT in presence of TGFβ. Both overexpression and knockdown studies have shown that FOXK1 plays a significant role in regulating TGFβ-induced EMT in colorectal cells [144].

Elevated level of FOXG1, has been reported to be associated with increased incidence of metastasis in human HCC. In fact, FOXG1 was found to play an essential role in HCC cells undergoing EMT. It was shown that FOXG1 activated Wnt signaling pathway through its association with β-Catenin and LEF1/TCF4, and thereby induced EMT [145].

Increased expression of FOXM1 has also been shown to significantly correlate with EMT marker proteins in tissue specimens from non-small cell lung cancer (NSCLC) patients. Subsequently, FOXM1 was reported to regulate EMT in the NSCLC cells through activation of AKT/p70S6K pathway [146]. FOXM1 was further shown to be induced by TGFβ in kidney cells undergoing EMT, where it was demonstrated to play a significant role [147].

Although the Forkhead box TFs as discussed above have been shown to regulate EMT in a positive manner, a few of the members of the family, such as FOXF2, FOXN2, and FOXO3a have been found to act as EMT suppressor [148–151]. FOXF2 has been shown to be a negative regulator of EMT in basal-like breast cancer cells and also in triple-negative breast cancer cells. Indeed, FOXF2 deficiency led to increased incidence of metastasis *in vivo* [148]. Mechanistically, FOXF2 was found to be directly recruited at the promoter region of EMT promoting TFs such as Twist1, FOXC2, and FOXQ1 and repress their transcription, and thereby suppress EMT [148,152,153]. Moreover, FOXF2 was shown to recruit nuclear receptor corepressor 1 (NCoR1) and HDAC3 to the FOXQ1 promoter to repress its transcription. FOXN2, on the other hand, repressed Slug expression by binding to its promoter directly and thereby inhibiting its transcription [149]. FOXO3a, another member of the Forkhead box family, has been reported to play a negative regulatory role during EMT in prostate cancer cells. It was shown to suppress the β-Catenin pathway by reducing its expression via activation of miRNA-34b/c, as well as by directly binding to β-Catenin, and thereby blocking the β-Catenin/TCF4 complex formation required for the activation of β-Catenin signaling pathway [150].

### SRY-related HMG-box family of TFs

SRY-related HMG-box 4 (Sox4), one of the members of the SRY-related HMG-box family TFs has been shown to play significant role during EMT in various cell types including mammary epithelial cells, breast cancer cells, gastric cancer cells, prostate cancer cells, renal cancer cells etc [154–160]. SOX4 expression level was enhanced in TGFβ-treated cells undergoing EMT and was shown to promote mesenchymal characteristics as a part of EMT. Notably, high SOX4 expression level was found to be significantly correlated with triple-negative breast cancer [154], breast cancer metastasis [155], as well as gastric cancer [160]. SOX4 was further demonstrated to directly bind to the upstream regulatory region of Polycomb-group histone methyltransferase EzH2 [155], metalloproteinase ADAM28 [161] and N-Cadherin [157], thereby activating their transcription. Significantly, EzH2 was further shown to be a critical regulator of EMT in mammary epithelial cells. Interestingly, Wang et al. [158], showed that the ETS transcription factor ERG, which is induced in prostate cancer cells undergoing EMT, binds to the SOX4 promoter directly and stimulates its transcription. Moreover, SOX4 was shown to interact with ERG itself and promote EMT in prostate cancer cells.

The TF, SOX9, was found to be required for neural crest development [162,163]. Moreover, it was also shown to be expressed during hepatic stellate cell activation and caused type I Collagen production in presence of TGFβ [164]. SOX9 has further been reported to promote EMT in various cancer types such as thyroid cancer [165], prostate cancer [166], NSCLC [167], and gastric cancer cells [168]. It was also shown to be elevated in TGFβ treated human oral squamous cell carcinoma cells [169]. Mechanistically, SOX9 was shown to activate the Hippo-Yap signaling pathway in gastric carcinoma cells and Wnt/β-Catenin pathway in NSCLC cells to induce the EMT process. SLUG was further shown to directly interact with SOX9, thus blocking its ubiquitin-mediated proteosomal degradation, and thereby stabilizing it [170].

Another SOX family member SOX11 has also been shown to play significant role during EMT in breast cancer cells. It was reported to directly bind Slug promoter and induce its expression [171]. Interestingly, Oliemuller et al. have shown that SOX11 could promote epithelial–mesenchymal hybrid characteristics in breast cancer cell population [172]. The authors further demonstrated that SOX11 activity towards EMT is partially mediated by a potential downstream effector molecule, MEX31.

### RUNX family transcription factors RUNX1/2

RUNX1 has been shown to play significant role during EMT in colorectal cancer cells [173,174]. Indeed, TGFβ treatment was found to induce RUNX1 expression in colorectal cancer cells as well as in kidney epithelial cells undergoing EMT [174,175]. Moreover, RUNX1 was shown to promote renal fibrosis by activating transcription of PI3K subunit p110δ [175]. Mechanistically, RUNX1 was found to activate the Wnt/β-Catenin signaling pathway to promote EMT. In renal tubular epithelial cells, RUNX1 was shown to promote TGF-β-induced partial EMT by activating transcription of the PI3K subunit p110δ, which mediated Akt activation [175]. Additionally, RUNX2 was also shown to play significant role during EMT in thyroid carcinoma, hepatocellular cancer, renal cell carcinoma, and NSCLC cells [176–178]. In fact, both overexpression and knockdown studies with RUNX2 were shown to perturb the EMT process. Furthermore, a specific RUNX2 isoform has been shown to play critical role in mediating EMT in the developing heart of chick embryo independent of Snail2 [179]. Significantly, RUNX2 was also shown to facilitate pulmonary fibrosis [180].

### GATA family TFs

Zhou et al., employed both overexpression and knockdown studies to demonstrate that GATA4 is an inducer of EMT in nasopharyngeal carcinoma cell line. They further showed that GATA4 activated SLUG transcription by directly binding to its promoter region [181]. Interestingly though, GATA4 was also shown to induce moderate MET in HCC cells and cellular senescence by activating NF-κB pathway [182].

Another member of the GATA family TF GATA6 has been shown to induce EMT in cholangiocarcinoma cells. Both overexpression and knockdown studies were shown to affect the EMT process. GATA6 was shown to up-regulate Mucin-1 (*MUC1*) gene, a membrane-bound glycosylated protein involved in forming protective mucous barrier on epithelial cells, by directly binding to its promoter region [183]. It was reported to promote EMT in breast cancer cells as well, where it was shown to bind SLUG promoter and stimulate its transcription [184]. Campbell et al., found that GATA-factor Serpent (Srp) in *Drosophila*, an ortholog of human GATA6 is essential for EMT in the *Drosophila* endoderm. The authors further showed that Srp suppresses crumbs (crb), an epithelial cell polarity and adherens junction regulator, by directly binding to its promoter. Moreover, they also showed that human GATA6, when ectopically expressed, can induce EMT in MDCK cells in a similar fashion [185]. Martinelli et al., on the other hand, found that GATA6 acts as an inhibitor of EMT in pancreatic cancer cells. The authors further showed that GATA6 directly bound promoter regions of E-cadherin and Vimentin and regulated their transcription [186]. Significantly, both GATA4 and GATA6 have been shown to play critical role in cell migration during gastrulation in *Xenopus* embryo.

**Wilms’ tumor 1** (**WT1**) protein has been shown to play crucial role during both EMT and MET in a tissue-dependent manner, thereby maintaining the epithelial–mesenchymal balance. Indeed, WT1 was reported to promote MET during kidney development, but also induced EMT during heart development. It was shown to activate Snail1 and repress E-Cadherin by directly binding respective upstream regulatory elements in epicardial cells and during ES cell differentiation [187]. WT1 was found to act as EMT promoter in ovarian cancer cells as well, where it was shown to bind to the E-Cadherin promoter and thereby suppress its expression. It also activated the ERK1/2 signaling pathway in the same context [188]. Notably, several reports also showed EMT opposing function for WT1 protein. Noortje et al. reported that WT1 expression was suppressed in human adult epicardial cells upon TGFβ exposure. Furthermore, knockdown of WT1 was also lead to induction EMT in epicardial cells, where WT1 was shown to bind to Slug promoter and suppress its activity [189,190]. Interestingly, Sampson et al. reported that WT1 could induce a hybrid epithelial–mesenchymal state in clear cell renal cell carcinoma, where it stimulated expression of epithelial markers, such as E-cadherin and at the same time up-regulated expression of Snail, an EMT promoter [191]. It should also be noted that WT1 was shown to function as a key regulator during mesothelial–myofibroblast and fibroblast–myofibroblast transformation, therefore highlighting its role during tissue fibrosis [192].

The homeobox TF, **Goosecoid**, has been demonstrated to induce EMT in both breast cancer cells and HCC cells [193,194]. In fact, Goosecoid expression was found to be elevated in breast epithelial cells undergoing EMT when exposed to TGFβ. It also increased the incidence of lung metastases in mice [193,194]. Significantly, high Goosecoid level correlated well with poor survival and increased lung metastases in HCC patients [194]. It should be noted that Goosecoid has been shown to regulate cell migration in *Xenopus* embryo during gastrulation [195].

The homeobox TF, **Six1**, was shown to be involved during EMT in human mammary carcinoma cells [196], colorectal cancer cells [197], immortalized human keratinocytes [198], cervical cancer [199] as well as in lung epithelial cells [200]. Wang et al. have further reported that the Six1 protein level goes up in lung epithelial cells undergoing EMT when exposed to TGFβ. Importantly, Six1 was shown to play a significant role during conversion of lung epithelial cells into fibroblasts and associated airway remodeling [201].

Paired-related homeobox 1 (**PRRX1**) has been found to induce EMT in gastric cancer cells, NSCLC cells as well as in salivary adenoid cystic carcinoma cells [202–204]. Ocana et al. showed that PRRX1 could induce full EMT in chicken embryo as well as in MDCK cell line. The authors further demonstrated that loss of PRRX1 is required for the cancer cells to metastasize, whereby the cells regain epithelial characteristics needed to form colonies at the secondary site [205]. Prrx1 was further shown to induce hepatic stellate cell movement during liver fibrosis [206]. Mechanistically, PRRX1 was shown to induce EMT by activating the Wnt/β-Catenin pathway in gastric cancer cells [202]. Interestingly, Zhu et al., observed that knockdown of PRRX1 led to induction of EMT in the lung cancer cell line A549. PRRX1-deficient A549 cells were further shown to acquire cancer stem cell-like properties [207].

**Elk3** has been shown to positively influence EMT in breast cancer cells as well as during progression of liver fibrosis [208–210]. Indeed, Elk3 expression was up-regulated in breast cancer cells and liver cells following TGFβ treatment. Subsequently, SMAD3 and ZEB1 both were found to directly regulate Elk3 expression by binding to its promoter region [209,210]. ZEB1 was further shown to form complex with Elk3 and suppress E-Cadherin expression [209].

The T-box TF, **Brachyury**, was shown to promote EMT in pancreatic cancer cell line and lung carcinoma cells [211]. Moreover, Brachyury expression was found to be correlated well with EMT as well as lymph node metastasis in oral squamous cell carcinoma [212]. Furthermore, Brachyury expression was also reported to be up-regulated in kidney cells undergoing EMT in presence of TGFβ [213]. It was suggested that Brachyury probably binds the half T-element present on the E-cadherin promoter and thereby suppresses its expression. In addition, Brachyury has been shown to be play significant role in cell movement during gastrulation as well as mesoderm formation [214]. It was also found to promote renal interstitial fibrosis [213].

### AP-1 transcription factor

Both **FOSL1** and **FOSL2** have been reported to promote EMT in prostate cancer cells and NSCLC cells [215,216]. FOSL1/FRA-1 was further reported to induce EMT in mammary epithelial cells through direct binding of the TGFB1 and ZEB2 promoters as well as the first intron of ZEB1 and thereby regulating their expression [217]. **JunB** was shown to be involved during EMT in mammary epithelial cells [218]. Here JunB expression was reported to be elevated in mammary epithelial cells undergoing EMT in presence of TGFβ. Moreover, JunB was found to suppress Id2 (inhibitor of EMT) expression in cooperation with ATF3, a basic leucine zipper protein. Gong et al. showed that JunB could play a key role in IL6-stimulated EMT and aggressiveness in uveal melanoma cells [219]. Additionally, JunB has been shown to promote EMT in human renal tubular cell line. Here, ETS2, a member of conserved TFs of the ETS family, was demonstrated to directly bind to the JunB promoter and enhance its transcription [220]. Notably, the m6A methyltransferase METTL3, was also shown to stabilize the JunB mRNA, in lung cancer cells undergoing EMT [221].

Roca et al. have shown that **OVOL1** and **OVOL2** act as critical regulators of MET in mesenchymal prostate cancer cells and triple-negative breast cancer cell line. They further showed that OVOL2 binds to ZEB1 promoter directly and suppresses its transcription [222]. Watanabe et al., have recently reported that OVOL2 could induce MET in fibroblasts in cooperation with tissue specific re-programming factors such as KLF4 and TP63 [223]. Zinc finger and BTB domain-containing 38 (**ZBTB38**) has been reported to promote EMT in bladder cancer cells by activating Wnt/β-Catenin signaling pathway [224]. The Aristaless-like homeobox1 (**ALX1**) or Cart1 TF has been shown to induce EMT in ovarian cancer cells and breast epithelial cells in a Snail1-dependent manner [225]. The TF, **TFAP2A**, expression was reduced in breast epithelial cells undergoing EMT in presence of TGFβ. It was further shown to negatively regulate the EMT process by directly binding to ZEB2 promoter and repressing its transcription [226]. Interestingly, deletion of TFAP2A has been shown to inhibit fibroblast to myofibroblast conversion, indicating its possible role during tissue fibrosis [227]. Zhao et al. have identified BTB Domain and CNC Homolog 1 (**BACH1**) TF as promoter of EMT in esophageal squamous cell carcinoma cells. They have shown that BACH1 directly binds to the promoter region of CDH2, SNAI2, Vimentin, and VEGFC genes and regulate their transcription [228]. BACH1 was further shown to induce EMT and promote pancreatic cancer metastasis (Sato et al., *Cancer Res.* (2020) **80**(6), 1279–1292). Significantly, inhibition of BACH1 has been found to attenuate bleomycin-induced lung fibrosis in mouse [229,230].

Recently, Meyer-Schaller et al. used siRNA-based, functional microscopy screen to identify 46 (co)transcription factors along with multiple miRNAs that were shown to play essential roles in mammary epithelial cells undergoing EMT in presence of TGFβ [231]. In this study, transcriptomics, interactome analysis, and computational analysis were done to reveal transcriptional regulatory networks regulating the EMT process in normal mouse as well as human mammary epithelial cells. Along with known EMT TFs, this study also identified few novel EMT TFs/co-regulators. Additionally, helicases such as DDX5, DDX20/DP103, and DHX9/DDX9 have also been implicated in cancer aggressiveness [232–234]. Phosphorylated form of DDX5 (p-DDX5) was shown to mediate EMT by activating the Wnt/β-Catenin pathway [235]. It has also been found to stimulate Snail1 transcription by facilitating HDAC1 dissociation from the Snail1 promoter [236]. On the other hand, ectopic expression of DDX20/DP103 has been shown to enhance invasive abilities of breast cancer cells. Furthermore, its expression was also found to be correlated with metastasis gene signature as well as breast cancer metastasis [232]. Moreover, combination of DDX20/DP103 along with Amphiregulin and Cyclin A1 has been correlated with aggressive forms of oral squamous cell carcinoma with up-regulated EMT-associated gene signature [234]. DHX9/DDX9 has been found to inhibit EMT in human lung adenocarcinoma cells via STAT3 modulation [237]. Additionally, it was also shown to modulate circulatory RNAs during EMT [238]. Ring1b, a core component of the PRC1 has been shown to form complex with DEAD-box RNA helicases such as DDX3X and DDX5 and down-regulate E-cadherin by binding to its promoter [239].

## Transcriptional regulatory network in EMT

From the above discussion, it is evident that EMT is regulated by many TFs. Some of these EMT TFs such as Snail, Zeb, and Twist can control the process in totality, whereas others such as FOXC1, FOXC2, and RUNX1 can control only part of it. Infact, few of the identified TFs such as SOX11 and WT1 were found to induce a hybrid epithelial–mesenchymal state, thereby creating a flexible, plastic situation within the cell. Significantly, expressions of all these EMT TFs have been found to be regulated in a spatiotemporal manner under both normal physiological and pathological conditions. As a result, their contribution towards EMT were found to vary depending upon the cell/tissue types involved as well as EMT context/types (Tables 1 and 2 and above sections). Indeed, some of these TFs such as SNAI1 and ZEB1 were shown to induce EMT in most of the tumor cell/tissue types tested, while few others such as GATA4, GATA6, PRRX1 etc. were found to promote EMT in certain cell/tissue types only, and even block the EMT process in others. For example, GATA4 has been shown to induce EMT in nasopharyngeal carcinoma cells, whereas it was found to induce MET in HCC cells. GATA6 has been shown to induce EMT in breast cancer cells, however it was found to suppress EMT in pancreatic cancer cells (Tables 1 and 2). Different phenotypes exhibited by various EMT TF mutants during embryonic development also suggest differential contribution of these EMT regulators towards EMT. Table 1 further shows that the master regulators of EMT such as Snail1, slug, Zeb1, Zeb2, and Twist1 play critical roles during EMT in all different contexts, such as EMT types I, II, and III. On the other hand, as shown in Table 2, very few of the other EMT TFs were shown to take part in all EMT contexts. In fact, most of these other EMT TFs such as KLF or FOX family members mentioned in the text/Table 2, were shown to be involved during EMT associated with the carcinogenesis process only (EMT type III). This suggests that the combination of EMT TFs involved in a specific context might vary. Significantly, along with common EMT-associated functions, these EMT TFs have also been found to exert non-redundant functions. Indeed, besides metastasis, EMT and therefore EMT-TFs have been shown to participate in other processes such as resistance to cell death and senescence, resistance to different types of therapy, immune regulation, acquisition of stem cell-like characteristics etc., that are important for carcinogenesis (type III EMT). In addition, EMT TFs are involved in different morphogenetic functions including left-right asymmetry regulation, bone morphogenesis, neural tube morphogenesis etc. Possible reason for this pleotropic behavior of EMT TFs include the nature of the EMT inducer(s) as well as the upstream signaling pathway(s) involved under different contexts. Importantly, different inducer and/or upstream signaling could also influence differential expression of factors other than EMT TFs, thereby altering interactome of the EMT TFs. The structural difference between different EMT TF families, and even within the same family members, as depicted in Figure 2, has been found to contribute towards the differential roles of these EMT TFs during EMT, as well. Additionally, all these EMT TFs undergo extensive post-transcriptional and post-translational modifications in a context-dependent manner, which could alter their interaction with specific partners, such as other TFs, co-regulators or epigenetic modifiers, thereby leading to specific outcome for each of these EMT TFs.

**Figure 4:**
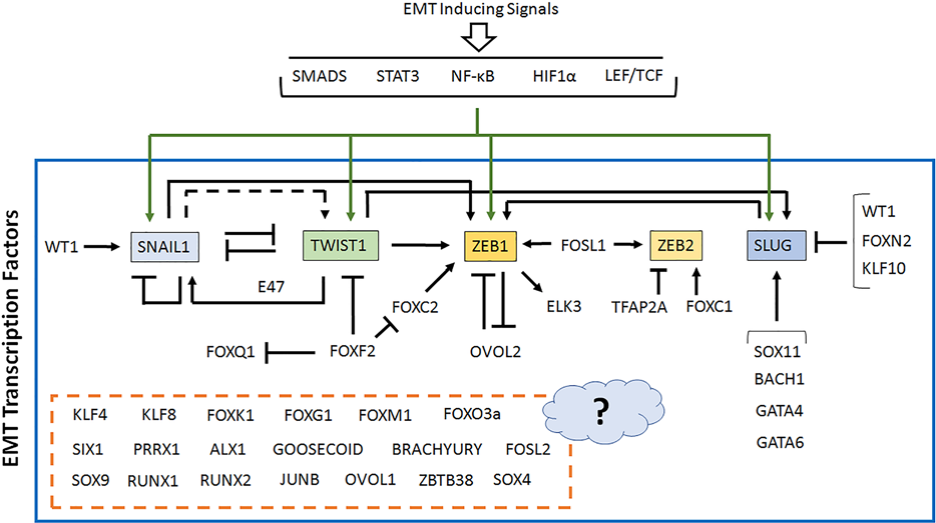
Transcriptional regulatory network in EMT EMT is regulated by many TFs, expression of which are dependent on each other to a large extent, thereby creating a complex transcriptional regulatory network. The master regulators of EMT are shown within colored boxes, while all other EMT TFs are shown as it is. For some EMT TFs, no information regarding their cross-regulation is known, so these are kept within a broken box attached with a question mark. The transcriptional regulatory network is derived from currently available literature (please see text for details) and include all different cell/tissue types as well as different EMT contexts.

Notably, expressions of these EMT TFs were found to be dependent on each other to a large extent (Figure 4). For example, SLUG expression was found to be positively regulated by EMT TFs such as SOX11, GATA4, GATA6 and BACH1, while KLF10, FOXN2 and WT1 was shown to suppress its expression in various cell/tissue types. Furthermore, SNAIL1, FOXC2, and TWIST1 all were found to induce ZEB1 transcription, whereas OVOL2 suppressed it. In fact, ZEB1 and OVOL2 were found to suppress each other’s transcription [240]. ZEB2 transcription was shown to be activated by FOXC1, FOSL1 and TFAP2A, while TWIST1 was shown to positively regulate transcription of ZEB1 [105] and SLUG [241]. Interestingly, TWIST1 and SNAIL1 appeared to share a complex relationship during EMT. For example, TWIST1 was shown to suppress SNAIL1 transcription when acted alone but activated SNAIL1 in presence of E47 [242]. SNAIL1 was further shown to directly suppress TWIST1 expression [48,49]. Dave et al., however, reported a positive effect of SNAIL1 on TWIST1 expression [243], although, it is not clear whether it was a direct effect or an indirect one. Some of these EMT TFs were also shown to target and induce expression of their co-transcription factors/binding partners, thereby creating a feed-forward mechanism to facilitate their own function. These types of interdependency in fact suggest presence of transcriptional hierarchy as well as temporal regulation within the transcriptional regulatory network behind EMT. Although, few of these TFs were reported to act alone and regulate transcription of target genes, some of them have also been found to collaborate with each other to regulate target gene expression. Furthermore, the upstream signaling pathways are also known to cross-talk with each other. At this point, it should also be noted that apart from the transcription factors described here, there are also a significant number of other co-transcription factors and/or epigenetic modifying enzymes along with non-coding RNAs including miRNA, which have been reported to exhibit EMT regulatory functions. Evidently, the transcriptional network regulating EMT has also been found to control expression of these co-transcriptional regulators and the non-coding RNAs during EMT.

All these observations suggest presence of a very complex, dynamic, and flexible transcriptional regulatory mechanism behind the EMT process, which probably functions in a context-dependent manner (EMT type I, II, or III). It also seems very likely that the transcriptional regulatory network controlling the EMT process in various cell/tissue types have both common and distinct elements in them.

## EMT TFs and their significance

**Figure 5:**
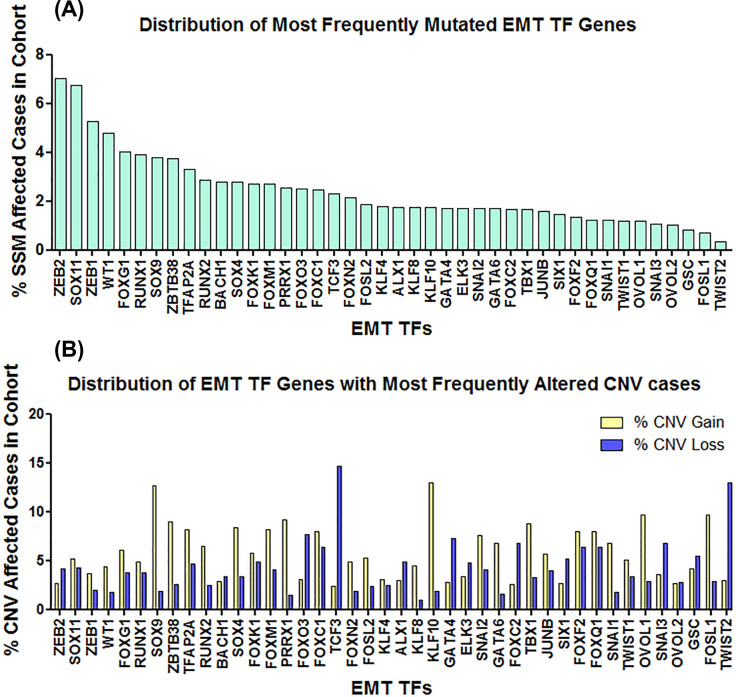
Genetic alterations of EMT TF genes TCGA data were analyzed for the presence of (**A**) SSMs and (**B**) CNV within the set of EMT TFs discussed in this review. Our analysis revealed ZEB2, SOX11, and ZEB1 to be the most frequently mutated EMT TFs (SSMs), whereas TCF3, TWIST2, KLF10, and SOX9 to be the EMT TFs with most frequently altered CNV cases.

Although, the master regulators of EMT such as Snail, Slug, Zeb, Twist have been shown potential as prognostic markers for various cancer types as discussed above, sufficient information regarding mutational status of these molecules are lacking. For that matter, we analyzed the data generated by The Cancer Genome Atlas (TCGA) and looked for presence of simple somatic mutations (SSMs) within the set of EMT TFs discussed in this review. Our analysis revealed that ZEB2 (7.01% of affected cases), SOX11 (6.75% of affected cases), ZEB1 (5.28% of affected cases), WT1 (4.8% of affected cases), and FOXG1 (4.04% of affected cases) are the most frequently mutated EMT TF genes (Figure 5A) in the cohort (data obtained from GDC portal) among our set of EMT TFs. Furthermore, ZEB2 was found to be most frequently mutated in endometrial carcinoma (16.23%); SOX11 in colon adenocarcinoma (28.75%), endometrial carcinoma (19.81%), and esophageal carcinoma (19.02%); ZEB1 in endometrial carcinoma (12.5%) and melanoma (11.1%); WT1 in acute lymphoblastic leukemia (16.1%); and FOXG1 in colon adenocarcinoma (11.25%). Besides, in these cases, all the EMT TFs were found to be mutated in several other cancer types at relatively higher frequency (Table 3). It is also interesting to note that most of the EMT TFs we analyzed, were found to be mutated at a significant frequency in both endometrial carcinoma as well as colon adenocarcinoma (Table 3). Further analysis of these EMT TFs for the presence of Copy Number Variations (CNVs) revealed significant changes in copy number for EMT TF genes such as TCF3 (% CNV gain 2.35, % CNV loss 14.7), TWIST2 (% CNV gain 2.97, % CNV loss 12.95), KLF10 (% CNV gain 13.01, % CNV loss 1.85), SOX9 (% CNV gain 12.71, % CNV loss 1.91), FOXQ1 (% CNV gain 8.0, % CNV loss 6.32), FOXF2 (% CNV gain 7.98, % CNV loss 6.33), and FOXC1 (% CNV gain 7.92, % CNV loss 6.33) etc (Figure 5B). Significantly, we have found CNV loss in 58.97% cases of ovarian serous cystadenocarcinoma and 51.97% cases of uterine carcinosarcoma for TCF3; CNV loss in 25.85% cases of cervical squamous cell carcinoma and endocervical adenocarcinoma, 22.31% cases of sarcoma, 21.2% cases of ovarian serous cystadenocarcinoma and 21.08% cases of bladder urothelial carcinoma for TWIST2; CNV gain in 29.74% cases of ovarian serous cystadenocarcinoma, and 20.59% cases of bladder urothelial carcinoma for KLF10; CNV gain in 27.52% cases of ovarian serous cystadenocarcinoma and 20.06% cases of breast invasive carcinoma for SOX9; CNV gain in 26.32% cases of ovarian serous cystadenocarcinoma for FOXQ1 and FOXF2; and CNV gain in 25.81% cases of ovarian serous cystadenocarcinoma for FOXC1 among others (Table 4). One caveat in our analysis using TCGA-derived data is that EMT status of the patient tissue samples are unknown. Nevertheless, the single somatic mutation and copy number variation analysis in this article clearly signifies the importance of these EMT TFs during the development and progression of cancer in specific cell/tissue types. Significantly, besides facilitating the metastatic dissemination of cancer cells, EMT has also been observed during early stages of tumorigenesis [244,245], therefore suggesting a wider role for these EMT TFs during cancer development.

**Table 3:** Percentage* of SSM affected cases in cohort (TCGA)

Projects	Study name	ZEB2	SOX11	ZEB1	WT1	FOXG1	RUNX1	SOX9	ZBTB38	TFAP2A	RUNX2	BACH1	SOX4	FOXK1	FOXM1	PRRX1
CMI-ASC	Angiosarcoma Project	5.56			11.1		5.56									
CMI-MPC	Metastatic Prostate Cancer				6.67											
MMRF-COMMPASS	Multiple Myeloma Commpass study		5.32													
TARGET-ALL-P3	Acute Lymphoblastic Leukemia Phase 3				16.1		7.14									
TARGET-WT	High-risk Wilms’ tumor				5.26											
TCGA-ACC	Adrenocortical carcinoma		7.61													
TCGA-BLCA	Bladder Urothelial Carcinoma	5.1														
TCGA-BRCA	Breast invasive carcinoma						5.07									
TCGA-CESC	Cervical squamous cell carcinoma and endocervical adenocarcinoma	6.57	8.3													
TCGA-CHOL	Cholangiocarcinoma															
TCGA-COAD	Colon adenocarcinoma	8.75	28.75	6.75	10.3	11.25		16		6.25			14.3	5.5		
TCGA-DLBC	Lymphoid Neoplasm Diffuse Large B-cell Lymphoma		13.51			5.41										
TCGA-ESCA	Esophageal carcinoma	7.61	19.02			5.43					5.43					
TCGA-KICH	Kidney Chromophobe		6.06													
TCGA-LAML	Acute Myeloid Leukemia				8.33		9.72									
TCGA-LIHC	Liver hepatocellular carcinoma		10.16													
TCGA-LUAD	Lung Adenocarcinoma	9.35		7.94												
TCGA-LUSC	Lung Squamous Cell Carcinoma	9.9		8.08	5.25	5.05										
TCGA-READ	Rectum adenocarcinoma	8.03	15.33	5.11		5.11		7.3					5.11			
TCGA-SARC	Sarcoma		7.17													
TCGA-SKCM	Skin Cutaneous Melanoma			11.1	5.76											
TCGA-STAD	Stomach adenocarcinoma	8.86		5.45		5.91			6.59		5.23					
TCGA-UCEC	Uterine Corpus Endometrial Carcinoma	16.23	19.81	12.5	9.81	8.87	12.08	11.1	13.02	10.57	8.3	11.51	10.8	8.3	7.55	8.11

*We have shown changes only 5% and above.

**Table 4:** Percentage* of CNV affected cases in cohort (TCGA)

Project	Study name	TCF3		TWIST2		KLF10		SOX9		FOXQ1		FOXF2		FOXC1	
		Gains	Losses	Gains	Losses	Gains	Losses	Gains	Losses	Gains	Losses	Gains	Losses	Gains	Losses
TCGA-BLCA	Bladder Urothelial Carcinoma			1.23	21.08	20.59	0.49	12.75	0.74						
TCGA-BRCA	Breast invasive carcinoma	1.68	14.74			18.66	1.12	20.06	2.05						
TCGA-CESC	Cervical squamous cell carcinoma and endocervical adenocarcinoma	2.72	14.97	0.68	25.85										
TCGA-CHOL	Cholangiocarcinoma					16.67	0								
TCGA-ESCA	Esophageal carcinoma	1.09	13.04	2.17	15.22	15.22	2.17	13.59	4.89	8.15	16.3	7.61	16.3	7.61	16.3
TCGA-HNSC	Head and Neck squamous cell carcinoma	0.77	11.13	1.34	17.47										
TCGA-LIHC	Liver hepatocellular carcinoma					13.75	0.27								
TCGA-LUAD	Lung Adenocarcinoma					11.89	2.14	10.53	0.78						
TCGA-LUSC	Lung Squamous Cell Carcinoma	1.99	11.75	1.79	19.12	14.94	1.59	15.14	1.39						
TCGA-OV	Ovarian serous cystadenocarcinoma	1.37	58.97	11.28	21.2	29.74	5.98	27.52	1.71	26.32	12.14	26.32	12.31	25.81	12.14
TCGA-SARC	Sarcoma	10	15	4.23	22.31			12.31	2.69						
TCGA-SKCM	Skin Cutaneous Melanoma							11.97	1.28	11.11	1.5	11.11	1.5	10.9	1.07
TCGA-UCEC	Uterine Corpus Endometrial Carcinoma	0.2	18.43												
TCGA-UCS	Uterine Carcinosarcoma	1.79	51.79			16.07	0	19.64	0	8.93	10.71			8.93	10.71
TCGA-UVM	Uveal Melanoma					15.19	0					8.93	10.71		

*We have shown changes of only 10% and above in either of a pair of Gain or Loss.

## EMT inhibitors as potential anti-cancer/anti-fibrosis drugs

**Figure 6:**
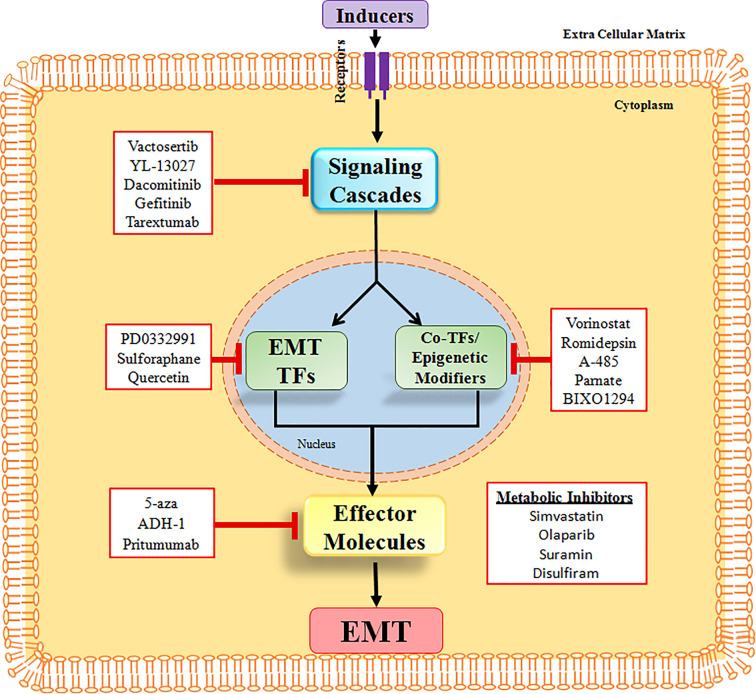
The EMT inhibitors and their targets Small molecule inhibitors are being used to suppress the EMT process. They target various key components of EMT, including the EMT TFs.

From the above discussion, it is quite evident that EMT is involved during various stages of carcinogenesis and is responsible for acquisition of different characteristics such as stem cell properties, chemoresistance, as well as resistance to immunotherapy by the cancer cells. Therefore, inhibiting the EMT process, in theory, is not only going to inhibit cancer cell dissemination and therefore inhibit metastasis, but it will also make the cancer cells more sensitive towards various forms of therapy. The strategies that are being used to target the EMT process involve: (a) Targeting extracellular inducers and signaling pathways of EMT, (b) Targeting EMT TFs and associated cofactors as well as epigenetic modifiers, (c) Using metabolic pathway inhibitors to block EMT, and (d) Targeting the mesenchymal cell-specific molecules (Figure 6) [246–248]. Many small molecules including derivatives of natural products as well as already approved drugs (being used for other purposes) have been shown to suppress EMT by targeting various mediators/pathways as per the above strategies. Significantly, these EMT inhibitors exhibited both anti-cancer and anti-fibrosis activity in multiple tissue types, suggesting they may be effective for both cancer patients and patients with fibrotic tissue.

It should be noted that although, these EMT inhibitors have shown great potential as anti-cancer/anti-fibrosis drug, several concerns still exist regarding their use in patients. In general, within a tumor at the primary site, only a very small number of tumor cells undergo EMT. This might increase the chance for these tumor cells undergoing EMT to escape the drug. Furthermore, cells are known to undergo transient and incomplete EMT process, thereby creating a spectrum of hybrid epithelial/mesenchymal states with great functional flexibility, which makes it difficult to target these cells. EMT was also shown to occur even during early tumorigenesis process. This early dissemination of tumor cells may lead to presence of tumor cells in circulation as well as at the secondary sites. Use of EMT inhibitors might induce the reverse process, MET, in these cells, and therefore could facilitate colonization at the secondary site. Inhibition of EMT in these cells might also increase their proliferation, which in turn could lead to rapid tumor growth in both primary and secondary sites. Although, this would make the target tumor cells vulnerable to common chemotherapeutic drugs targeting rapidly dividing tumor cells. Significantly, the key players involved in EMT, such as the inducers, signaling pathways, even the EMT TFs are also known to be involved in other processes such as normal stem cell functioning, immune response etc. Furthermore, EMT is also known to facilitate the wound healing process. Consequently, EMT inhibitor therapy might elicit serious side effects in patients. Taken together, it is clear that a better understanding of the EMT process, especially the hybrid state, as well as more preclinical data regarding response to potential EMT inhibitors are required to develop a workable therapeutic strategy.

## Conclusion and perspectives

Work from various laboratories have been able to identify a large group of TFs regulating EMT in various cell/tissue types as well as in different contexts. We are only beginning to understand how all these transcription factors as well as other co-transcriptional regulators and non-coding RNAs act together to form a dynamic regulatory network in a context-dependent manner to control the transition. Evidently, controlled expression of pro- and anti-EMT TFs, or even certain EMT TFs alone, seems to have the potential to create an epithelial–mesenchymal hybrid state with maximum plasticity, thereby keeping the cell in an optimal state, so that it can initiate its movement from the primary location. Notably, most of these EMT transcription factors were studied in isolation in a cell/tissue type-specific manner (Tables 1 and 2). Consequently, we still do not have enough information regarding the relationships/cross-talks that exist among them in different cell/tissue types or even within the same cellular context. As a result, when we talk about the transcriptional regulatory network responsible for EMT, we currently have fragments of information coming from different sources. Furthermore, for a number of these EMT TFs, we still do not know anything about their direct targets (Table 2). Consequently, a far more in-depth study of EMT in a cell/tissue type as well as context-dependent manner is needed to have a better understanding of the process.

## Abbreviations

bHLH: basic–helix–loop–helix
CtBP: C-terminal-binding protein
DNMT: DNA Methyl Transferase
EMT: epithelial–mesenchymal transition
HCC: hepatocellular carcinoma
HDAC: histone deacetylase
KLF4: Krüppel-like factor 4
MET: mesenchymal–epithelial transition
NSCLC: non-small cell lung cancer
PRC: Polycomb Repressive Complex
PRRX1: paired-related homeobox 1
SOX: SRY-related HMG-box
TCGA: The Cancer Genome Atlas
TF: transcription factor
WT1: Wilms’ tumor 1
Zeb: zinc finger E-box binding
